# Comprehensive Evolutionary Analysis of Lamprey TNFR-Associated Factors (TRAFs) and Receptor-Interacting Protein Kinase (RIPKs) and Insights Into the Functional Characterization of TRAF3/6 and RIPK1

**DOI:** 10.3389/fimmu.2020.00663

**Published:** 2020-04-15

**Authors:** Jianqiang Hou, Yue Pang, Qingwei Li

**Affiliations:** ^1^College of Life Sciences, Liaoning Normal University, Dalian, China; ^2^Lamprey Research Center, Liaoning Normal University, Dalian, China

**Keywords:** TRAF family, RIPK family, evoluation, lamprey, immune regulation

## Abstract

TNFR-associated factors (TRAFs) and receptor-interacting protein kinases (RIPKs) are important immunological linker molecules in mammals and play important roles in the TNFα, TLR and IFN signaling pathways. However, the evolutionary origins of these genes in vertebrates have not previously been described in lampreys. In this study, we searched the genomes of *Lampetra japonicum, Lethenteron reissneri*, and *Petromyzon marinus* for genes encoding *trafs* and *ripks* and performed homologous sequence alignment, phylogenetic tree, functional domain, conserved motif, gene structure, and synteny analyses to determine their evolutionary relationships. The distribution of the lamprey *traf* and *ripk* families and the immune response of the gene families in lampreys stimulated by different pathogens were also demonstrated, suggesting a role of structural changes in expression and functional diversification. Additionally, the dual luciferase reporter gene assay showed that the addition of exogenous immunomodulator (TNFα or IFN) to the overexpression of LjLRIPK1a or LjTRAF3/6 significantly downregulated NF-κB or ISRE activation. LjRIPK1a can significantly enhance caspase-8 activity, and overexpression of LjRIPK1a or LjTRAF3a/6 in HEK293T cells results in cell apoptosis. In summary, this study makes an important contribution to the understanding of the *traf* and *ripk* gene families in different vertebrates. Our results also provide new evidence for the evolution of vertebrate TRAFs and RIPKs and their impacts on immune regulation.

## Introduction

Tumor necrosis factor receptor-associated factor (TRAF) protein is a key adaptor molecule linking immune receptor and intracellular signal transducers, contributing to various aspects of immune response and development processes. Currently, there are seven family members (TRAF1-TRAF7) identified in mammals. These proteins are involved in a variety of receptor-mediated cellular signaling pathways, including tumor necrosis factor receptor (TNF-R), interleukin-1 receptor, Toll-like receptor (TLR), nucleotide-binding domain and leucine-rich-repeat (NLR), and retinoic acid-inducible gene-1-like receptor (RLR) (1–4). In addition, these proteins also play important roles in the immune response, cell death and survival, development, and thrombosis. The identified TRAF molecules (except TRAF7) have a MATH-TRAF domain at the C-terminus. This domain can, in turn, be subdivided into a C-terminal β-sandwich domain (TRAF-C) and an N-terminal coiled-coil region (TRAF-N), which use TRAF-C domain to recognize receptors and TRAF-N domain for signaling (1, 3). TRAF1 has a Zn-Finger domain in addition to the conserved MATH-TRAF domain. TRAF2, TRAF3, TRAF5, and TRAF6 are highly consistent in domain composition, including a MATH-TRAF domain, a RING domain, and a different number of Zn-Finger domains. In addition, only TRAF4 contain a nuclear localization signal. TRAF7 possess a RING domain and a Zn-Finger domain in structure compared to the other TRAFs, and a characteristic domain of 4-7 WD40 repeat at the C-terminus. The present research has focused on the function of TRAF2, TRAF3, and TRAF6 in inflammation, immunity, and tumors in humans. The TRAF gene family has been reported from invertebrates to vertebrates, such as bivalvia (5, 6), cephalochorda (7), pisces (8–13), aves (14–16), and mammalia (7).

The receptor-interacting protein kinase (RIPK) family members are important sensors for intracellular and extracellular stress. These proteins have been shown to play important roles not only in inflammation and immune responses but also in the induction of programmed death (17–19). The RIP kinases now contain seven members, all of which share a homologous kinase domain but have different functional domains (17). RIPK1, the “founding member” of the family, was initially identified as a death domain (DD)-containing protein that interacts with the DD of the death receptor CD95 (Fas) (20). RIPK1 has an amino-terminal kinase domain, a carboxy-terminal DD and a bridging intermediate domain (ID) that contains a RIP homotypic interaction motif (RHIM). RIPK2 contains a carboxy-terminal caspase-activation-and-recruitment domain (CARD). RIPK3 also has an RHIM but lacks a DD. The RHIM domain likely mediates protein–protein interactions, since it is required for the interaction between RIPK3 and RIPK1 (21, 22). RIPK4 and RIPK5 are characterized by ankyrin repeats at the C-terminus. RIPK6 (LRRK1) and RIPK7 (LRRK2) have leucine-rich repeats, ankyrin repeats, Ras (GTPase) of complex proteins (ROC), and carboxyl terminus of ROC (COR) domains. Current research indicates that RIPK1 and RIPK3 kinase activities have been linked to the process of regulated necrotic cell death or “necroptosis”. However, few studies have linked inflammation and immunity to the function of RIPK4-RIPK7 (19). Some studies have also shown that RIPK6 and RIPK7 play important roles in Parkinson's disease (23–25). Current results demonstrate that the members of each family of RIPKs are ubiquitous and highly conserved in vertebrates. However, genes containing the RIPK1 domain structure can be found in the genomes of echinoderms and phylum hemichordata, as well as the cephalochordate subphylum and urochordata subphylum. They are usually present in multiple copies. The RIPK3 gene only appears in vertebrates but is presently unidentified in cephalochordata and urochordata (26).

At present, lamprey has received attention in the fields of comparative genomics based on its primitive genome. Accumulating genomic data provide clear evidence for gene numbers, genome duplications and time of vertebrate origins during the evolution of vertebrates. Hence, lamprey is a useful model organism for the study of multigenic families. Here, a comprehensive survey and evolutionary analysis of lamprey TRAF and RIPK families was performed to illuminate key aspects of their evolution. Moreover, the study of lamprey TRAF and RIPK families is of great significance not only to reveal the new functions of this complex defense system in this primitive species but also to characterize the evolution and development of TRAF-mediated signaling and RIPK-induced signaling in higher vertebrates.

## Materials and Methods

### Animal Maintenance

Adult lamprey (*Lampetra japonica)* (30–50 cm in length), including males and females, were obtained from the Tongjiang Valley of Songhua River, Heilongjiang Province, China. These lampreys were maintained in 200-L tanks of a recirculating system at 10 ± 2°C at Liaoning Normal University for two weeks prior to challenge and RNA isolation. Animals were sacrificed in 0.01% MS-222 followed by exsanguination. Adult lamprey blood was collected by cutting the tail, and leukocytes were separated by Ficoll-Paque gradient centrifugation of the blood with lymphocyte separating solution (160 × g, 20 min) (27–29). Subsequently, the lamprey lymphocytes were collected for the following studies. All studies were reviewed and approved by the Animal Welfare and Research Ethics Committee of the Institute of Dalian Medical University's Animal Care protocol (Permit Number: SCXK2008-0002).

### Sequence Alignments and Analyses of Phylogeny and Conserved Motifs

The amino acid sequences of RIPKs and TRAFs involved in sequence alignments and phylogenetic analysis were obtained from the NCBI (http://www.ncbi.nlm.nih.gov/). Then, sequence alignments were performed using Clustal W, and the aligned sequences were used to construct rooted phylogenetic trees with the neighbor-joining (NJ) method using MEGA X software with the pairwise-deletion option (30–32) (N-J: Test of Phylogeny: Bootstrap method; Replications: 1000; Method: Poisson model; Gaps: Pairwise deletion; ML: Test of Phylogeny: Bootstrap method; Replications: 1000; Method: Jones-Taylor-Thornto (JTT) model; Gaps: Pairwise deletion.). The analysis of motifs was predicted by MEME (http://meme-suite.org/tools/meme) with the following parameters: zero repetitions per sequence or one repetition per sequence, a motif size between 6 and 50 aa, and a maximum of 20 motifs (33).

### Structural Analysis

The exon–intron organization of RIPK and TRAF genes was obtained from Ensemble (http://asia.ensembl.org/index.html). The protein structural domains were predicted by NCBI (https://www.ncbi.nlm.nih.gov/Structure/cdd/wrpsb.cgi) and InterPro (http://www.ebi.ac.uk/interpro/). Then, the domain maps are drawn by IBS software.

### Synteny Analysis

Genomic synteny was obtained from Genomics v92.01 (http://www.genomicus.biologie.ens.fr), Ensembl (http://asia.ensembl.org/index.html), and the Medical Research base (SIMRbase, https://genomes.stowers.org/). To facilitate cross-species comparisons, neighboring genes of the *ripk* and *traf* genes from the human, mouse, chicken, zebrafish, and hagfish genomes were used as reference genomic regions. For instances of uncertain identity (e.g., genes annotated with numerical identifiers), similarity searches were conducted to establish possible homology relationships between genes.

### Quantitative RT-PCR

The 54 lampreys were equally allocated to 18 groups, separately immunized with *Vibrio anguillarum* (1 × 10^7^ cells/fish), *Staphylococcus aureus* (1 × 10^7^ cells/fish), and poly I:C (100 μg/fish) by intraperitoneal injection, and sacrificed at 2, 8, 24, 48, and 72 h post-immunization. The PBS group was used as a control group. Then, leukocyte RNA was extracted and reverse-transcribed with a PrimeScript RT-PCR kit (TaKaRa, China) (34). Importantly, the genomic DNA was removed according to the instructions to eliminate interference. cDNA was used as a template to determine RNA expression of the *traf* and *ripk* family genes. qPCR was performed in triplicate using a TaKaRa PCR Thermal Cycler Dice Real Time System, and *L-gapdh* (GenBank accession No. KU041137.1) was used as an internal control (28, 29, 34). The reaction efficiency was tested by the gradual dilution of cDNA template (1x, 5x, 10x, 20x, and 40x). It was confirmed that the amplification efficiency of all primers was between 0.9 and 1.1. And the specificity of the amplification reaction was analyzed by dissociation curve analyses. The primers for qPCR are shown in Table S1.

### Coimmunoprecipitation

HEK293T cells (1 × 10^7^ cell/mL) in 10-cm dishes were transfected with 10 μg DNA plasmids (pEGFP-N1-LjRIPK1a and pcDNA3.1-HA-LjTRAF3a/6). At 28 h posttransfection, whole cell extracts were prepared in 1 mL RIPA buffer (Beyotime) and a mixture of protease inhibitors (Beyotime). Experiments were performed on whole cell lysates using mouse anti HA monoclonal antibodies and GFP monoclonal antibodies (Abcam, Cambridge, UK) to confirm that the protein was expressed. Samples were precleared by incubating with 30 μL protein G–agarose suspension and isotype antibody for 30 min followed by centrifugation at 12,000 × g for 1 min to remove the beads. The precleared lysates were incubated with 30 μL of the protein G–agarose suspension and HA monoclonal antibody (2 μg/1 mL) on a rotator for 12 h at 4°C. Protein G–agarose beads were centrifuged at 12,000 × g for 12 s and washed three times with 1% Nonidet P-40 lysis buffer. Samples treated with 2-ME were separated on 12% SDS-PAGE gels, transferred onto nitrocellulose membranes (Millipore), and immunoblotted with GFP monoclonal antibodies (2 μg/1 mL).

### Dual-Luciferase Reporter Assay

The pEGFP-N1-LjRIPK1a and pcDNA3.1-HA-LjTRAF3a/6 plasmids were constructed and extracted using the TAKARA MidiBEST Endo-free Plasmid Purification Kit, and the concentration was determined to be more than 0.5 μg/μL. HEK293T cells (1 × 10^6^ cell/mL) were plated in 96-well plates and transfected 24 h later with a mixture of DNA by using Lipofectamine 3000 (Invitrogen). The mixed DNA contained 10 ng/well RL-TK vector, 100 ng/well NF-κB or ISRE vector and complementary empty vectors. The pNF-κB-Luc plasmid, pISRE-Luc plasmid, and pRL-TK-luc plasmid were purchased from Promega. The empty vector was used as the negative control group, and the positive control group used the immunomodulator concentrations as follows: LPS, 2 μg/μL; TNFα, 2 μg/μL; and IFN, 2 μg/μL. After 24 h of transfection, the immune modulator concentration was added for stimulation for 6 h. Then, samples were detected using SpectraMax i3 using 560 and 465 nm wavelengths, respectively, according to the manufacturer's instructions (Beyotime, RG027).

### Apoptosis Assay

HEK293T cells (1 × 10^6^ cell/mL) were plated in 96-well plates and transfected with 150 ng/well indicated plasmids. pEGFP-N1, and pcDNA3.1 were used as a control group. After 48 h of transfection, staining was performed using Annexin-V Alexa Fluor 555 or Annexin-V-FITC. The nucleus was stained with Hoechst. Stain at 37°C in the dark for 10 min. The high content screening system (PerkinElmer) was then used for testing, and the results were counted by Harmony4.1.

### Caspase-8 Activity Assay

HEK293T cells (1 × 10^6^ cell/mL) were plated in 96-well plates and transfected with 150 ng/well indicated plasmids. The negative control group used the same quality pEGFP-N1 plasmid. The positive control group was stimulated with 2 μg/mL TNF-α for 4 h. At 20 h after transfection, the Caspase-Glo 8 assay system (Promega) was tested using SpectraMax i3 according to the manufacturer's instructions (scanning at full wavelength).

### Statistical Analysis

All statistical analyses were performed using GraphPad Prism 7.0 software. Differences between treatment groups were determined by two-way ANOVA. *P* < 0.05 was set as the threshold for significance (^*^P < 0.05, ^**^P < 0.01, ^***^P < 0.001, ^****^P < 0.0001; ^#^*P* < 0.05, ^##^*P* < 0.01, ^###^*P* < 0.001, ^####^*P* < 0.0001 and ^+^*P* < 0.05, ^++^*P* < 0.01, ^+++^*P* < 0.001, ^++++^*P* < 0.0001).

## Result

### TRAF Family in Lampreys

**Figure 1:**
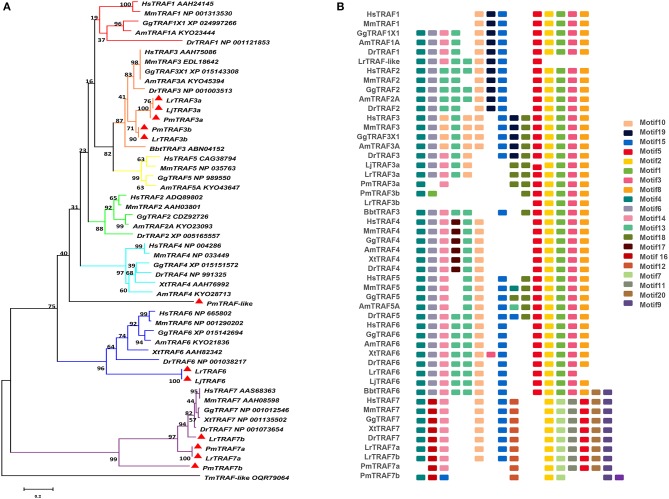
Phylogenetic relationships and architecture of conserved protein motifs in TRAF genes from lampreys. **(A)** The phylogenetic tree was constructed based on the full-length sequences of vertebrate TRAF proteins using MEGA X software. **(B)** The motif composition of lamprey TRAF proteins. The motifs, numbers 1–20, are displayed in different colored boxes. The sequence information for each motif is provided in Table S3. Hs: *Homo sapiens*; Mm: *Mus musculus*; Am: *Alligator mississippiensis*; Bbt: *Branchiostoma belcheri tsingtauense*; Dr: *Danio rerio*; Gg: *Gallus gallus*; Xt: *Xenopus tropicalis*; Lj: *Lampetra japonicum*; Lr: *Lethenteron reissneri*; Pm: *Petromyzon marinus*; Tm: *Tropilaelaps mercedesae*. The GenBank accession numbers of the lamprey sequences submitted in this study are recorded in Table S2.

The TRAF gene family was found to have three members in Drosophila. In humans, the family has evolved into seven molecules with various functions. Moreover, there are seven TRAFs in sea urchins, eight in zebrafish, and 24 members in amphioxus (15–18). By conducting similarity searches and homology inferences from the currently available genome and transcriptome data, we identified twelve *traf* genes in the genome of sea lampreys (*P. marinus*), five *traf* genes in the Japanese lampreys (*Lampetra japonicum*) transcriptome, and ten different *traf* sequences in the transcriptomes of reissner lamprey (*Lethenteron reissneri*). Finally, only six sequences and four members of the lamprey TRAF family, including *traf3, traf6, traf7* and *traf-like* were available and identified (Table S2). We used these sequences and the TRAF families from other species for a phylogenetic analysis based on their full-length amino acid sequences (Table 1). The phylogenetic tree showed two main groups, including the TRAF1-6 family and TRAF7 family. The lamprey TRAFs are located in the outer group of the vertebrate TRAF family. This finding is in line with the evolutionary status of each species. It is worth noting that there are two subtypes of lamprey TRAF3 and TRAF7, which are not shown in lower amphioxus or higher vertebrates. This property may indicate that the two genes may be a local tandem duplication in lamprey. Furthermore, TRAF1, TRAF2, TRAF4, and TRAF5 in the lamprey were unidentified, but there was a sequence in the outer cluster of TRAF1-TRAF5, known as TRAF-like. This gene may be derived independently of the common ancestor. TRAF1-TRAF5, which undergoes functional differentiation during subsequent evolution, eventually evolves the functions of multiple family genes (Figure 1A and Figure S1A).

**Table 1:** Summary of the sequence analysis of the *traf* gene family.

**Gene name**	**Species**	**Location**	**Gene length(kb)**	**Transcript length(bp)**	**No of exon**	**Amino acids(aa)**
*traf2*	Human	Chromosome 9	40.09	2266	11	501
	Mouse	Chromosome 2	28.96	3044	11	501
	Chicken	Chromosome17	16.59	1841	11	544
	Xenopus	Scaffold GL173718	9.44	1491	15	496
	Zebrafish	Chromosome 5	12.16	1791	10	575
	Zebrafish	Chromosome 5	28.75	1829	11	532
	Lamprey	Scaffold 00179	17.40	1357	4	452
*traf3*	Human	Chromosome 14	134.02	7700	11	543
	Mouse	Chromosome 12	100.69	7060	12	567
	Chicken	Chromosome 5	43.19	2004	12	567
	Xenopus	Scaffold GL172968	32.73	1686	10	561
	Zebrafish	Chromosome 17	21.76	2465	11	573
	Lamprey	Scaffold_00029	3.64	1692	13	563
		Scaffold 00036	4.26	1743	14	580
*traf6*	Human	Chromosome 11	21.08	2558	8	522
	Mouse	Chromosome 2	23.23	6169	8	530
	Chicken	Chromosome 5:	17.52	2372	7	545
	Xenopus	Scaffold GL172917	21.92	2760	7	558
	Zebrafish	Chromosome 7	18.86	4707	7	542
	Lamprey	Scaffold 00002	7.71	1767	6	589
*tra7*	Human	Chromosome 16	22.35	3683	21	670
	Mouse	Chromosome 17	10.36	2384	19	629
	Chicken	Chromosome 14	28.62	2811	21	670
	Xenopus	Scaffold GL172663	24.5	3526	21	666
	Zebrafish	Chromosome 3	26.25	3410	21	639
	Lamprey	Scaffold 02500	33.21	2347	20	776
		Scaffold 00037	35.94	2576	20	801

To identify structural features in the TRAF proteins, MEME was used for motif searching, and sequence logos of motifs 1–20 were drawn to determine the conserved amino acid residues (Figure 1B and Table S3). Five motifs (2, 4, 6, 8, and 20) appeared in almost all TRAF protein sequences. In the first group (TRAF1-6), 11 motifs (1, 2, 3, 4, 5, 6, 7, 8, 9, 17, and 20) were conserved, and some members had unique motifs, such as TRAF4 with motif 18, TRAF2/5 with motif 14 and TRAF6 without motif 10. TRAF7, as the second group, has a motif structure distinct from that of other TRAFs. In addition to having common motifs (2, 4, 6, 8, and 20), TRAF7 also has a unique motif structure (11, 12, 14, 15, and 16). By comparing the composition of motif and domain, we find that there is a correspondence between motif and domain. Motif4 and motif6 participate in the formation of the RING domain. Motif 10, 13, and 14 are involved in the formation of the Zn-Finger domain. Motif 12, 15, 18, or 19 are related to the coiled-coil domain. Motif1, 2, 3, 5, and 8 are involved in the formation of the MATH-TRAF domain. In addition, the formation of the unique WD40 domain of TRAF7 is linked to motif1, 5, 7, 9, 11, and 20. Compared to other vertebrates, the motif composition of the lampreys TRAF3 and TRAF7 is absent.

### Gene Structure Comparison of Lamprey TRAFs With Their Counterparts in Vertebrates

**Figure 2:**
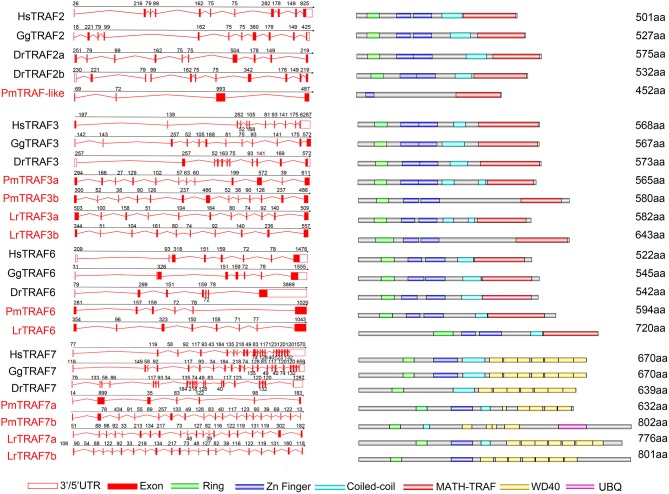
Genomic structure of TRAFs and diagrams of their protein domain structure in different classes of vertebrates. Comparison of vertebrate TRAF3, TRAF6, and TRAF7 gene structure. Different colored boxes represent various structures: blank boxes, 3' or 5' UTR; red line, introns; red boxes, exons. Different colors denote distinct domains: green, Zn ring; blue, Zn finger; red, MATH/TRAF and azure color, coiled-coil.

To further reveal the changes in the structure and function of vertebrate *trafs* during evolution, we further analyzed the genetic structure changes of the lamprey and other vertebrates. Comparison of orthologous exons and introns of *traf* from different species showed that exons and introns of *traf-like* and *traf3* had more significant differences than those of *traf6* and *traf7* during evolution (Table 1). The *traf-like* has only 4 exons compared to other vertebrate *traf2*, and two of the longer exons may be split into shorter exons during evolution (Figure 2). A comparison of functional domains reveals that vertebrate TRAF molecules are conserved in the functional domain. As shown in Figure 2, TRAF-like, TRAF3, and TRAF6 all have the following domains: RING, Zn-Finger, coiled-coil, and MATH-TRAF. Interestingly, there are two coiled-coils in lamprey TRAF3, while the other vertebrates have only one coiled-coil. The domain organization of lamprey TRAF7 is similar to that of other vertebrate TRAF7 proteins. TRAF7 has a RING domain and a Zinc-Finger domain in structure compared to other TRAF molecules, but the C-terminus is composed of a 4-7 WD40 repeat domain, which is different from the TRAF2/3/6 family.

### Syntenic Analysis of Lamprey *trafs*

**Figure 3:**
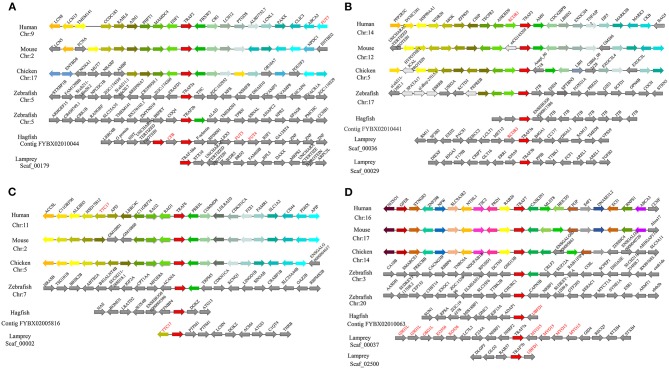
Syntenic relationship analysis of the TRAF family. The conservation of genes neighboring *traf2* **(A)**, *traf3* **(B)**, *traf6* **(C)**, and *traf7* **(D)**. Orthologous genes contributing to conserved synteny are similarly color-coded.

To gain insight into the evolutionary history of the *traf* gene family during the vertebrate radiation, the neighboring gene environment of lamprey *trafs* were compared with other vertebrate *trafs*. A chromosome region with a similar gene repertoire to that flanking human *trafs* was found in the mouse and chicken. In the zebrafish genome a chromosome region, homologous to the gene environment flanking the lamprey *trafs* has been lost from fish genomes. Interestingly, both zebrafish and hagfish contain two *traf2* copies on the same chromosome. The difference is that the two *traf2*s of zebrafish are far apart, while the two *traf2*s of hagfish are separated by only the *cfb* gene. In addition, *fut3* and *fut4* in the vicinity of hagfish *traf2* belong to the same gene family as *fut7* in the vicinity of human *traf2* (Figure 3A). The syntenic analysis of *traf3* revealed that both lamprey *traf3a* and human *traf3* orthologs are closely linked to members of the *rcor* family, indicating a close evolutionary relationship (Figure 3B). For syntenic analysis of *traf6*, the *ttc17* gene surrounding lamprey *traf6* also appeared in the adjacent genes of human, mouse, and chicken *traf6* (Figure 3C). Moreover, both zebrafish and lamprey contain two *traf7* molecules, but the two molecules are located on different chromosomes. Surrounding lamprey *traf7b, ubfd*1 gene is also found in the neighborhood of hagfish *traf7* gene. In particular, there are tandem repeats of *myo15, ko536* and *gsg1l* in the adjacent genes of lamprey *traf7a* (Figure 3D).

### RIPK Family in Lampreys

**Figure 4:**
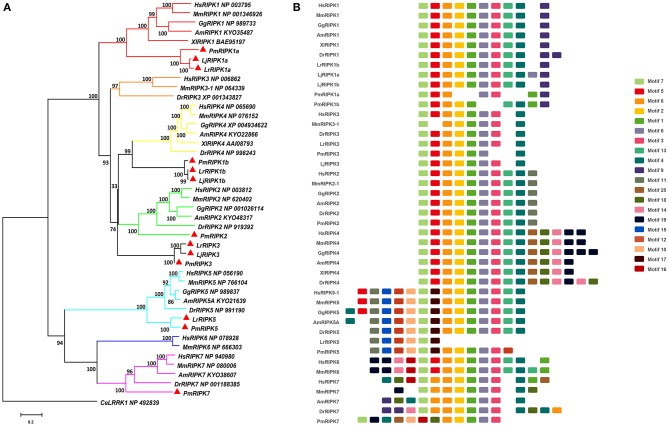
Phylogenetic relationships and architecture of conserved protein motifs in RIPK genes from lampreys. **(A)** Phylogenetic comparison of RIPKs in vertebrates. **(B)** The motif composition of RIPKs proteins. The sequence information for each motif is provided in Table S4. Hs: *Homo sapiens*; Mm: *Mus musculus*; Am: *Alligator mississippiensis*; Xl: *Xenopus laevis*; Bbt: *Branchiostoma belcheri tsingtauense*; Dr: *Danio rerio*; Gg: *Gallus gallus*; Lj: *Lampetra japonicum*; Lr: *Lethenteron reissneri*; Pm: *Petromyzon marinus*; Ce: *Caenorhabditis elegans*.

Based on genomic analysis, associated with the BLAST search and PFAM prediction domain method, we obtained five *ripk* family members related to *ripk1a, ripk1b, ripk2, ripk3, ripk5*, and *ripk7*. Then, the lamprey RIPKs and the other vertebrate RIPK family were used to construct a phylogenetic tree (Figure 4A and Table 2) based on their full length using MEGA X. Next, we constructed the NJ phylogenetic tree and the ML phylogenetic tree to analyze the phylogenetic relationships between the lamprey and other vertebrate RIPKs to better understand their origin and evolution. Two different phylogenetic trees comprise two main clades, representing RIPK1-4 and RIPK5-7 (Figure S1B). In addition to RIPK1b and RIPK3, all members of the evolution tree are classified into their respective branches. Lamprey RIPK1a, RIPK2, RIPK5, and RIPK7 form a cluster with its counterpart from other species and are located outside the vertebrate RIPK1, RIPK2, RIPK5, and RIPK7 clusters, respectively, which is consistent with the laws of biological evolution. This property suggests that these molecules are evolutionarily related to the ancestral genes of the corresponding molecules in vertebrates. RIPK1b is always located outside RIPK4 in two evolutionary trees, indicating that RIPK1b is derived directly from the common ancestor of the vertebrate RIPK4. The lamprey RIPK3 cannot be classified with other vertebrate RIPK3, suggesting that it may have functional uniqueness. To further determine the classification and evolutionary relationships of RIPKs, we analyzed the motifs of these proteins (Figure 4B and Table S4). The RIPK family has a conserved kinase domain; therefore, these proteins have a relatively conserved motif structure (motifs 1, 2, 3, 4, 6, 7, and 8). In addition, the unique domain of each molecule of the family shows the difference in the composition of its motif. RIPK1 has motif 9 at the C-terminus; RIPK3 does not have motif13; RIPK2 has motif 11 at the C-terminus; motifs 20, 18, 14, and 19 are at the C-terminus of RIPK4; and motifs 11, 15, 12, and 10 are at the N-terminus of RIPK5. Here, we found that a unique motif constitute an important domain. For example, the DD domain is related to motif9, and the CARD domain consist of only motif11.

**Table 2:** Summary of sequence analysis of the *ripk* gene family.

**Gene name**	**Species**	**Location**	**Gene length(kb)**	**Transcript length(bp)**	**No of exon**	**Amino acids(aa)**
*ripk1*	Human	Chromosome 6:	46.76	4160	11	671
	Mouse	Chromosome 13	31.32	3266	11	658
	Chicken	Chromosome 2	20.22	2793	12	658
	Xenopus	Scaffold GL172847.1	15.39	2010	10	667
	Zebrafish	Chromosome 2	16.22	2194	11	657
	Lamprey	Scaffold00020	20.04	2232	17	743
		Scaffold00008	10.986	1629	17	542
*ripk2*	Human	Chromosome 8	33.25	2516	11	540
	Mouse	Chromosome 4	40.91	2692	11	539
	Chicken	Chromosome 2	18.44	2673	11	574
	Xenopus	Scaffold GL173088	26.12	989	7	553
	Zebrafish	Chromosome 2	29.75	1598	10	584
	Lamprey	Scaffold00020	45.96	2118	20	705
*ripk3*	Human	Chromosome 14	3.97	1872	10	518
	Mouse	Chromosome 14	3.87	1892	10	486
	Xenopus	Scaffold GL172951	8.83	1275	10	514
	Zebrafish	Chromosome 7	18.14	1881	8	433
	Lamprey	Scaffold00008	7.25	909	8	303
*ripk5*	Human	Chromosome 1	69.06	7739	12	929
	Mouse	Chromosome 1	49.40	6282	13	927
	Chicken	Chromosome 26	20.04	3271	13	930
	Xenopus	Scaffold GL172677	37.50	3174	14	916
	Zebrafish	Chromosome 22	33.26	2825	13	857
	Lamprey	Scaffold GL477765	35.7	2355	10	788
*ripk7*	Human	Chromosome 12	144.29	9239	51	2527
	Mouse	Chromosome 15	142.95	8275	51	2527
	Chicken	Chromosome 1	64.09	9528	50	2557
	Xenopus	Scaffold GL172656	94.81	7530	56	2509
	Zebrafish	Chromosome 25	61.71	9170	51	2532
	Lamprey	Scaffold00010	34.71	5661	30	1473

### Analysis of the Lamprey RIPK Gene Structures

**Figure 5:**
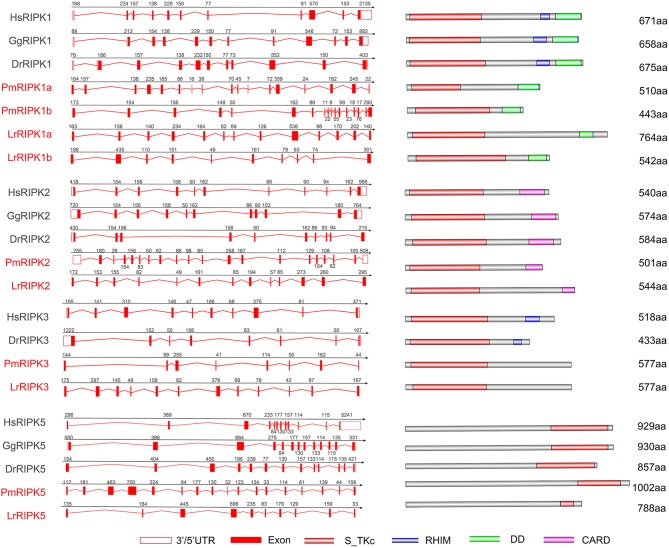
Genomic structure of RIPKs and diagrams of their protein domain structure in different classes of vertebrates. Comparison of vertebrate RIPK1, RIPK2, RIPK3, RIPK5, and RIPK7 gene structure. Different colored boxes represent various structures: blank boxes, 3' or 5' UTR; red line, introns; red boxes, exons. Different domains were drawn by colored boxes: red, S_TKc; blue, RHIM; green, DD and orange, CARD.

Analysis of exons/introns showed a significant difference between the seven *ripks* and other vertebrates (Figure 5 and Table 2). For example, lamprey *ripk1a* and *ripk1b* have 17 exons, while other vertebrates have only 11 exons. Lamprey *ripk2* has 20 exons, which is notably more than other vertebrates. In addition, the comparison of the functional structure of the lamprey RIPKs also shows the difference. Lamprey RIPK1 is divided into two distinct subtypes (RIPK1a and RIPK1b) with S_Tkc and DD domains similar to other vertebrates, but the lamprey RIPK1a and RIPK1b did not contain the RHIM domain. Interestingly, homologous sequence alignments reveal that lamprey RIPK1a has a conserved site IQIG similar to other RIPK1. This site mediates binding with the RHIM domain. Moreover, both lamprey RIPK1a and RIPK1b have a conserved site of DFG, indicating that both of them can be inhibited by Necrostatin-1 (Nec-1) (Figure S2). Similarly, Lamprey RIPK3 does not show the presence of the RHIM domain. Domain prediction results for lamprey RIPK2 demonstrate the S_Tkc domain and the CARD domain, while RIPK5 only has a relatively conserved S_Tkc domain.

### Syntenic Analysis of Lamprey *ripks*

**Figure 6:**
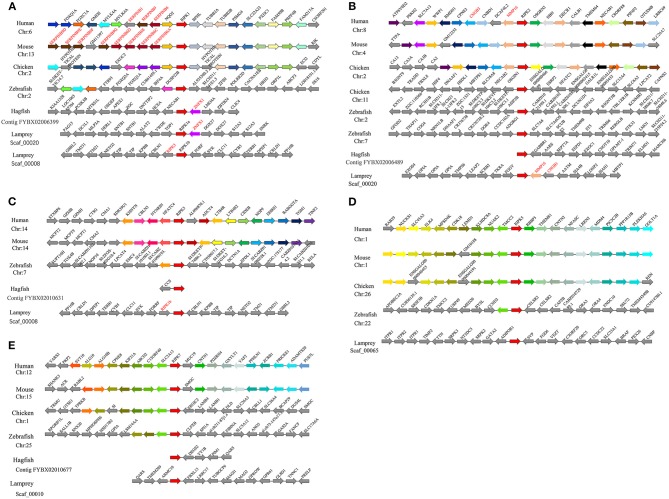
Syntenic relationship analysis of the RIPK family. The conservation of genes neighboring *ripk1* **(A)**, *ripk2* **(B)**, *ripk3* **(C)**, *ripk5* **(D)** and *ripk7* **(E)**. Orthologous genes contributing to conserved synteny are similarly color-coded.

Proximal genes from different species can provide information about phylogenetic relationships among members of a gene family. We conducted a comparative genomics study that performed a linear analysis of the adjacent genes of lamprey and other vertebrate *ripks*. In higher vertebrates, the orthologous genes of *ripks* remain in a certain conserved state, and the upstream and downstream genes of *ripks* are largely consistent. In lower vertebrates, there are more differences between the upstream and downstream genes of *ripks*. Moreover, we also found that there are a large number of *serpin* family members near *ripk1* in higher vertebrates. Two different *ripk1* molecules (*ripk1a and ripk1b*) exist in lamprey, in which the ortholog *znfx1* of *ripk1a* is also present in hagfish. More interestingly, lamprey *ripk1a* is located in the same scaffold_00020 as *ripk2*, but not in close proximity (Figures 6A,B). The positional gene *mmp16* of lamprey *ripk2* has a genetic inversion compared to higher vertebrates. The neighboring gene *cngb1* of lamprey *ripk2* was found its family member-*cngb3* on the periphery of other higher vertebrate *ripk2* (Figure 6B). Lamprey *ripk3* is located in the same scaffold_00008 as *ripk1b* (Figure 6C). Particularly, the loss of *ripk3* gene in bird suggests potentially a consequence of lineage specific gene deletions. The gene environment of *ripk5* and *ripk7* in lamprey and other vertebrates demonstrates a considerable difference, which indicates that the two genes may have more dramatic changes during the evolution process (Figures 6D,E).

### Expression Profiles of Lamprey *trafs* and *ripks*

**Figure 7:**
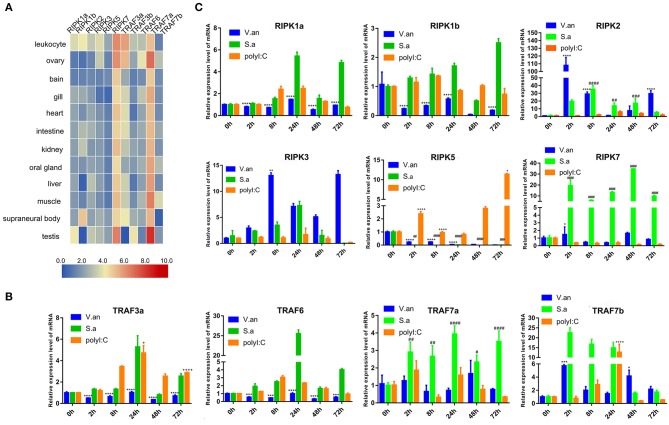
Expression profiles of lamprey RIPK and TRAF family genes. **(A)** Expression of *ripk* and *traf* genes in lamprey tissues. Original data from the database, data value represent reads per kilobase of transcript per million (RPKM). Q-PCR analysis of *trafs* **(B)** and *ripks* **(C)** in lampreys to characterize mRNA expression levels in leukocytes at 0, 2, 8, 24, 48, and 72 h after treatment with *V. anguillarum, S. aureus* and Poly I:C. All of the data are presented as the means ± SDs based on three independent cDNA samples with three replicates per sample. The asterisks indicate significant differences (*n* = 3, **P < 0.01 and *P < 0.05) compared to the control. The * represents statistical comparison between *V. anguillarum* stimulation group and control group. The # represents statistical comparison between *S. aureus* stimulation group and control group. The + represents statistical comparison between Poly I:C stimulation group and control group.

To verify the tissue distribution of *trafs* and *ripks* in lampreys, we examined their transcriptional levels in normal tissues (Figure 7A). In normal tissues, although the tissue expression levels of these two family molecules are different, they are mainly expressed in immune tissues or cells. In addition to *traf7a*, the remaining *traf* molecules are expressed in low amounts in various tissues and are mainly distributed in immune-related tissue cells, such as leukocytes, supraneural body and intestine. Lamprey *traf7a* is highly expressed in all tissues and is expressed most strongly in the testis. The expression distribution of the lamprey *ripk* molecules is similar to that of *trafs*. Lamprey *ripk1a, ripk1b, ripk2, ripk3*, and *ripk5* are expressed at low levels in all tissues and are mainly expressed in leukocytes. In addition, *ripk1a* is highly expressed in the testis, while *ripk1b* is highly expressed in the supraneural body. It is worth noting that *ripk7* has a high level of expression in all tissues and is considerably higher than other member molecules. To determine the role of *trafs* and *ripks* in the lamprey immune response, we also examined the effects of different pathogen challenge on them by qRT-PCR. Our data showed that lamprey *traf3a, traf6* and *traf7a/b* expression showed similar upregulation in response to Gram-positive bacteria and viral mimics. The difference may be that the transcription level of *traf7a* was significantly downregulated after 72 h of viral mimic stimulation. However, in response to Gram-negative bacteria, the four molecules showed significant differences. Transcription levels of lamprey *traf3a* and *traf6* showed a downward trend; in contrast, *traf7b* showed an upregulation trend, and *traf7a* showed no significant change (Figure 7B). These results suggest that lamprey *trafs* are involved in different signal regulation by regulating different levels of transcription in response to diverse stimuli. The stimuli response of the lamprey *ripk* molecule to three different immunogens showed significant differences in their stimulation of Gram-negative bacteria. Transcription levels of lamprey *ripk1a* and *ripk3* showed an upward trend, with the difference being that *ripk1a* returned to normal levels after 72 h of stimulation. Lamprey *ripk1b* and *ripk5* showed the same regulatory tendency, and transcriptional levels were inhibited. Lamprey *ripk7*, which has the highest transcription level in the normal state, has no observable response to Gram-negative bacteria. After stimulation with Gram-positive bacteria, transcription in lamprey was significantly upregulated, except for the inhibition of *ripk5* transcription levels. After viral mimic stimulation, the transcription of lamprey *ripk1a, ripk1b, ripk5*, and *ripk7* was upregulated, while *ripk7* transcription was markedly downregulated (Figure 7C).

### Functional Characterizations of Lamprey TRAF3a/6 and RIPK1a

**Figure 8:**
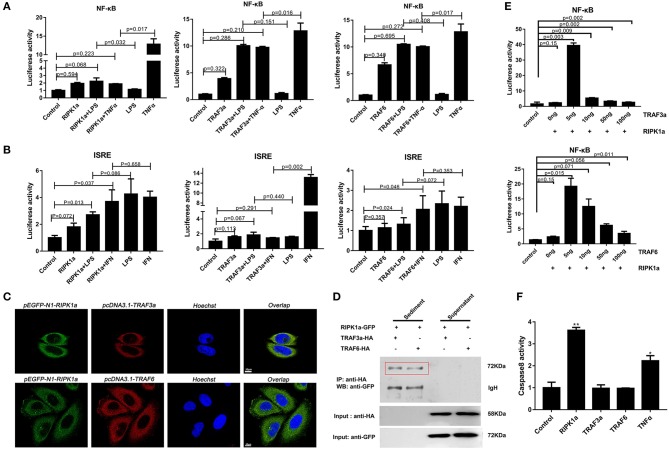
Effects of overexpression of RIPK1a, TRAF3a and TRAF6 on NF-κB, ISRE and caspase-8. **(A)** The effect of LjRIPK1a and LjTRAF3a/6 overexpression on NF-κB. HEK293T cells were cotransfected with 50 ng pEGFP-N1 empty vector or the indicated expression vector together with 100 ng corresponding luciferase reporter vector and 2 ng control Renilla expression vector. All reporter assays were performed in triplicate and repeated with three separate experiments. Values are expressed as the mean fold induction ± SD relative to that of the empty vector control from one representative experiment. **(B)** The effect of LjRIPK1a and LjTRAF3a/6 overexpression on ISRE. **(C)** Immunofluorescence microscopy images of HeLa cells cotransfected with GFP-fused LjRIPK1a with HA-tagged pcDNA3.1 LjTRAF3a or LjTRAF6 and stained with anti-HA and Alexa Fluor 555 Ab. Data are representative of at least three independent experiments in which 80% of the cells showed similar staining patterns. **(D)** Co-immunoprecipitation results show that LjRIPK1a interacts with LjTRAF3a and LjTRAF6 in HEK293T cells. **(E)** The effect of cotransfection of LjRIPK1a and LjTRAF3a/6 on NF-κB. **(F)** Overexpression of LjRIPK1a activates caspase-8. The positive control used human TNFα (2 ng/mL). Caspase-8 assays were performed in triplicate and repeated with three separate experiments. Values are expressed as the mean fold induction ± SD relative to that of the empty vector control from one representative experiment. Statistical significance is indicated (n = 3, **P < 0.01 and *P < 0.05).

In mammals, RIPK1 plays important roles in NF-κB and MAPK activation (17–21). Luciferase assays were performed to reveal whether LjRIPK1a has similar activities. Owing to the absence of lamprey cells currently, human cell lines were selected to express lamprey genes. Overexpression of LjRIPK1a, LjTRAF3a, and LjTRAF6 alone weak activated NF-κB or interferon-stimulated response element (ISRE) without statistical significant difference. Further, whether overexpression of LjRIPK1a or LjTRAF3a/6 in HEK293T cells by the stimulation of TNFα can inhibit NF-κB activation, as compared with normal HEK293T cells only by TNFα stimulation (Figure 8A). Similar results showed that neither the overexpression of LjRIPK1a nor LjTRAF6 to exogenous LPS or IFN stimulation showed an effect on ISRE activity. However, overexpression of LjTRAF3a significantly decreased ISRE activity through IFN stimulation (Figure 8B). In amphioxus, RIPK1a and RIPK1b directly interact with TRAF2, 3 and 6 to activate NF-κB (35). Thus, we further carry out co-expression of LjRIPK1a with LjTRAF3a or LjTRAF6 in the HeLa cells. Immunofluorescence results showed that LjTRAF3a, LjTRAF6 and LjRIPK1a were all localized in the cytoplasm (Figure S3). The colocalization results show that LjRIPK1a can colocalize with LjTRAF3a or TRAF6, respectively (Figure 8C). In addition, co-immunoprecipitation results demonstrate that LjRIPK1a can directly interact with LjTRAF3a and LjTRAF6 (Figure 8D). Based on the results of colocalization and interaction, we performed further analysis to assess the functional relevance between LjRIPK1a and LjTRAF3a/6. As shown in Figure 8E, lamprey RIPK1a strongly increased NF-κB activation induced by LjTRAF3a/6 through using luciferase reporter assays. Thus, we can assume that the primitive role of LjRIPK1a is involved in NF-κB activation dependent of LjTRAF3a/6, but not effect on ISRE activity. Importantly, cells overexpression LjRIPK1a were found to exhibit loss of adherence and membrane blebbing morphologically similar to apoptosis. Similar morphology was observed when HeLa cells were transfected with LjTRAF3a or LjTRAF6. Our results of the apoptosis assay by high content screening analysis reveal that overexpression of LjRIPK1a, LjTRAF3a, and LjTRAF6 induce cell apoptosis, and a histogram showing the statistics of the above mentioned results is shown in Figures S4, S5. Furthermore, we investigated whether the effect of LjRIPK1a, LjTRAF3a and LjTRAF6 overexpression on apoptosis depended on caspase-8 activity. Overexpression of LjRIPK1a significantly enhanced the activity of caspase-8, whereas LjTRAF3a and LjTRAF6 had no significant effect on the activity of caspase-8 (Figure 8F).

## Discussion

As TRAF molecules are important immunological molecules, they have been identified in both invertebrates and vertebrates, such as mussels (5), pearl oysters (6), fish (8–13), chickens (15), and pigeons (14, 16). All of the TRAF protein family members TRAF1 to TRAF7 are presented in zebrafish (36). In this paper, we identified and analyzed all of the TRAF molecules in lampreys and compared them to other vertebrates. TRAF1, TRAF2, TRAF4, and TRAF5 are present in zebrafish, but no predictions exist for these genes in lampreys. However, we found that lamprey TRAF-like gene is located in the outer group of other vertebrates TRAF1 to TRAF5 families, which is derived independently of the common ancestral gene. Moreover, both lamprey TRAF3 and TRAF7 have multiple copies. In contrast, for lower invertebrates, such as sea urchins, fish, and higher vertebrates, each TRAF family molecule exists in a single copy. Interestingly, TRAF4 is duplicated only in zebrafish, whereas several duplication events have occurred in the fish lineage for TRAF2 (36). In general, a complex genome contains more members of the gene family to increase complexity and functional specificity, suggesting that the expansion of the TRAF3 and TRAF7 lineages in lamprey may be due to new functional requirements. Thus, it is speculated that the TRAF protein family is subjected to various selection pressures in the evolutionary process, finally leading to the needs arising from the emergence of new molecules and the new functions generated.

Current research on the RIPK family focuses on the role of RIPK1-3 in the immune process, but little has been reported on the evolution of this family. We identified five members of RIPK family in lampreys. Among them, RIPK1 exists in two different subtypes and is located on the same scaffold as RIPK2 and RIPK3. Based on these data, we speculate that RIPK2 and RIPK3 can evolve from RIPK1 through structure-specific recombination. In addition, we also found a tandem repeat event in RIPK7, indicating that lamprey has undergone small-scale intrachromosomal gene duplications. In combination with the emergence of RIPK7 in invertebrates, we speculate that the RIPK7 molecule may be derived independently from the common ancestor genes. It is worth mentioning that both the lamprey RIPK1 and RIPK3 genes lack RHIM domains with a key binding site for two molecules, mediating programmed cell necrosis in higher vertebrates. Deletion of this RHIM domain in the lamprey indicates that different signaling pathways may be present to participate in the immune response. In sum, for the TRAF family and RIPK family, clear orthologies exist between lamprey and mammalian lineages, with a few duplications for some of the gene family members.

Lamprey *traf* family members is preferentially expression in immune-related tissues, such as leukocytes, supraneural body and intestines. Whereas *ripk1a* expression is highest in the testis, and *ripk1b* is expressed predominately in the supraneural body, suggesting that they may have differential roles in different tissues. It has been reported that the transcription level of TRAF3 is increased after Gram-negative bacterial stimulation, and TRAF6 is first increased and then decreased in large yellow croaker. However, after polyI:C stimulation, TRAF3 showed an upward trend, and TRAF6 showed a downward trend (10). In addition, the mRNA expression of TRAF3 is first increased and then reduced in sea cucumbers after receiving Gram-negative bacteria or LPS stimulation (10). TRAF3 was significantly elevated in chickens after polyI:C stimulation (15). Different tissue distributions and stimuli responses of lamprey TRAFs and RIPKs indicate that they participate in different immune responses in the species.

**Figure 9:**
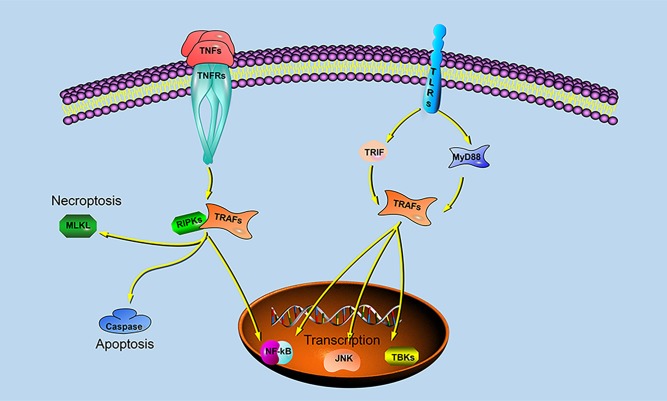
TRAF- and RIPK-mediated signaling pathways in lampreys.

Previous studies have shown that different species of TRAFs and RIPKs have different stimuli responses to different pathogens, which may be closely related to their living environment. To assess whether the endosome localization of LjRIPK1a, LjTRAF3a and LjTRAF6 was indispensable for its signaling activity, luciferase reporter assays were performed. LjRIPK1a, LjTRAF3a, and LjTRAF6 overexpression affected NF-κB activation. Similarly, overexpression of RIPK1 and TRAF6 in amphioxus also affected NF-κB activation, while TRAF3 showed no effect on the activation of NF-κB (35). In addition, overexpression of LjRIPK1a and LjTRAF6 resulted in a significant increase in ISRE expression levels. However, the addition of IFN after overexpression of LjTRAF3a significantly decreased the expression of ISRE compared to the group receiving only IFN treatment. It has been reported that different truncated mutants of TRAF3 have different effects on the activity of NF-κB, but no research has been reported on other TRAFs (5, 6, 16, 17). Future studies are required to elucidate the mechanism of TRAF3a/6 or RIPK1a interactions. In addition, our results demonstrate that overexpression of lamprey RIPK1a, TRAF3a and TRAF6 induces cell apoptosis, and apoptosis caused by LjRIPK1a is associated with caspase-8, which was consistent with the results of amphioxus (35, 37). Whereas the overexpression of TRAF6 in grouper can inhibit virus-mediated apoptosis (12, 13), suggesting that orthologous gene in different species may be involved in different signaling pathways and have different biological activities. In fruit flies, TRAF mediates the Eiger-Wengen-JNK and Toll-MyD88-Dorsal pathways for immune defense (38). In vertebrates, TRAFs are involved in TNF pathway and TLR pathway, and mediate induction of apoptosis, antiviral response and NF-κB activation (36, 39, 40). Recent accumulating evidences demonstrate that TRAFs and RIPKs have been shown to be involved in the TLR signaling pathway and TNF signaling pathway. Currently, key adapters, signal transducers and caspases in the TNF and TLR signaling pathways have been identified in lampreys, and similar to vertebrate TNF and TLR signaling are also present in lampreys (Figure 9). In summary, our study is critical to elucidate the form of TNF and TLR systems in lamprey, providing fundamental background for further insight into the innate immune system of lamprey.

## Data Availability Statement

The datasets generated for this study can be found in Genbank, with the accession numbers: MN764208, MN764209, MN764210, MN764211, MN764212, MN764213, MN764214, MN764215, MN764216, MN764217, MN764218, MN764219, MN764220, MN764221, MN764222, MN764223, MN764224, MN764225, MN764226, MN764227, MN764228, MN764229, MN764230, MN764231, MN764232, MN764233, MN764234.

## Ethics Statement

The animal experiments were performed in accordance with the regulations of the Animal Welfare and Research Ethics Committee of the Institute of Dalian Medical University's Animal Care protocol (Permit Number: SCXK2008-0002).

## Author Contributions

QL, YP, and JH designed experiments. JH completed the experiment and analyzed the results. JH and YP wrote the manuscript.

### Conflict of Interest

The authors declare that the research was conducted in the absence of any commercial or financial relationships that could be construed as a potential conflict of interest.

## Supplementary Material

Figure S1 Phylogenetic relationship of the TRAFs **(A)** and RIPKs **(B)** in the vertebrate was constructed using the maximum likelihood (ML) method by Mega X with 1000 bootstrap replications.

Figure S2 Multiple alignments of L-RIPK1a/b with other known RIPK1 proteins in vertebrates. Important sites are circled by red squares. DL/FG can serve as a binding site for Nec-1. I/VQIG plays an important role in mediating protein-protein interactions with RHIM domains.

Figure S3 Immunofluorescence microscopy images of HeLa cells transfected with GFP fusion LjRIPK1a, HA-tagged pcDNA3.1-LjTRAF3a or -LjTRAF6 and stained with anti-HA and Annexin-V Alexa Fluor 555 Ab. Data are representative of at least three independent experiments in which >80% of the cells showed similar staining patterns.

Figure S4 Overexpression of LjRIPK1a leads to apoptosis. All reporter assays were performed in triplicate and repeated with three separate experiments. Values are expressed as the mean fold induction ± SD relative to that of the empty vector control from one representative experiment.

Figure S5 Overexpression of LjTRAF3a/6 leads to apoptosis. All reporter assays were performed in triplicate and repeated with three separate experiments. Values are expressed as the mean fold induction ± SD relative to that of the empty vector control from one representative experiment.

Table S1 Primers used in this study.

Table S2 GenBank accession number for lamprey sequences submitted in this study.

Table S3 Conserved motifs discovered among the amino acid sequences of the TRAFs from vertebrates using the MEME system.

Table S4 Conserved motifs discovered among the amino acid sequences of the RIPKs from vertebrates using the MEME system.
