# Supplementary figures and images for: Comprehensive Evolutionary Analysis of Lamprey TNFR-Associated Factors (TRAFs) and Receptor-Interacting Protein Kinase (RIPKs) and Insights Into the Functional Characterization of TRAF3/6 and RIPK1
Source: Front Immunol. 2020 Apr 15;11:663. doi: 10.3389/fimmu.2020.00663 (PMC7179693; doi:10.3389/fimmu.2020.00663)

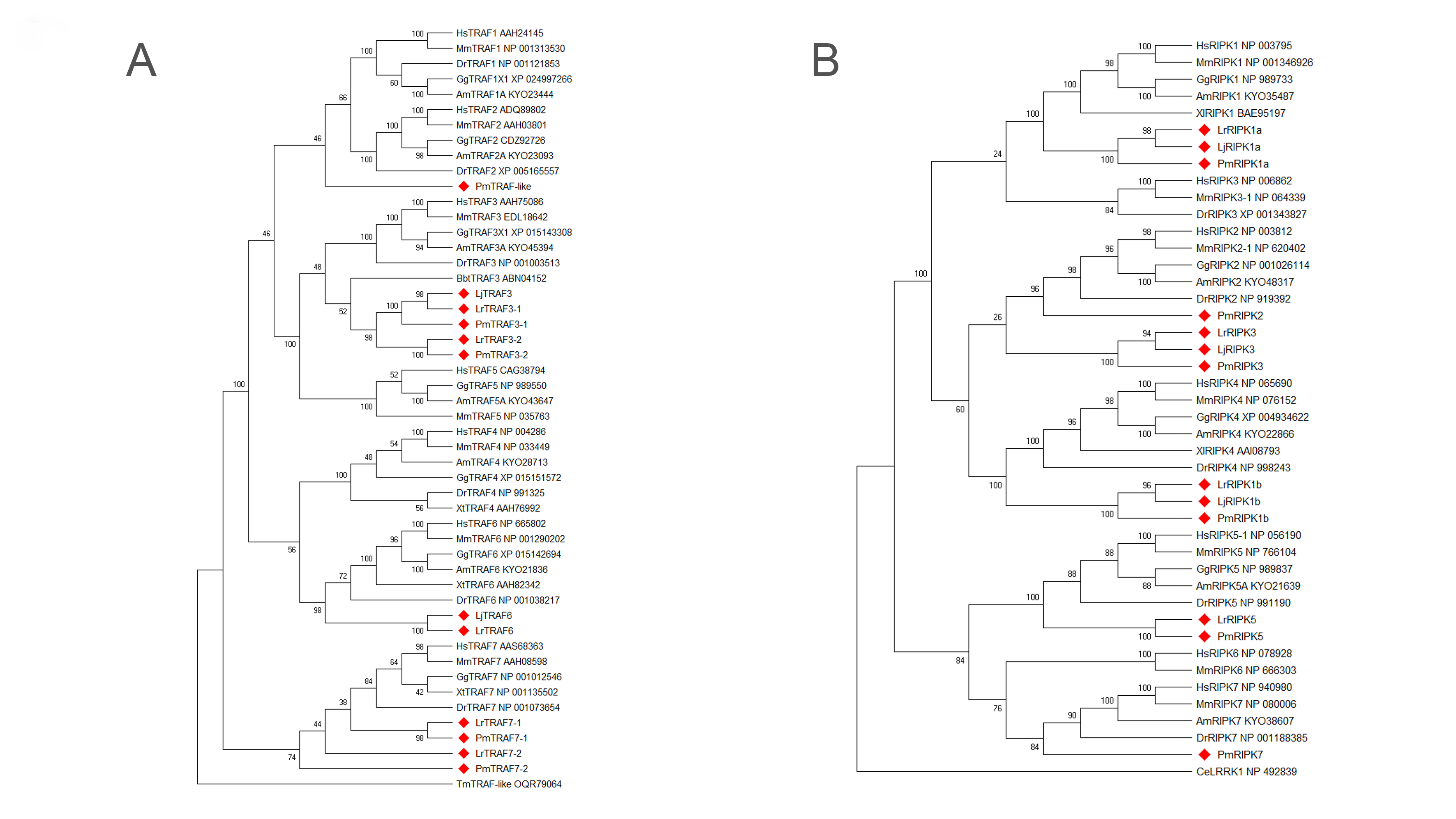

Supplement: Figure S1 — Phylogenetic relationship of the TRAFs (A) and RIPKs (B) in the vertebrate was constructed using the maximum likelihood (ML) method by Mega X with 1000 bootstrap replications. [file Image_1.TIF]

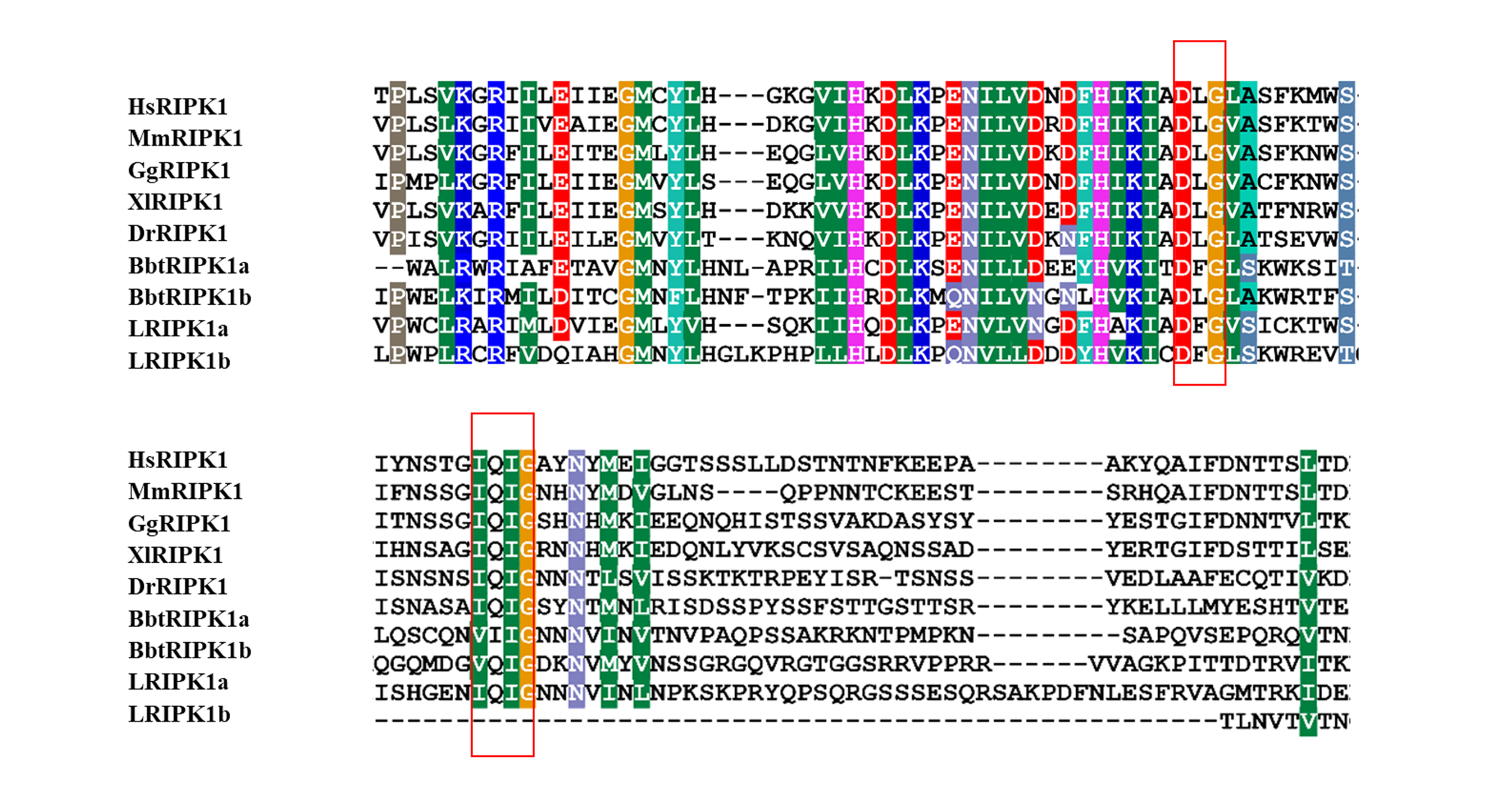

Supplement: Figure S2 — Multiple alignments of L-RIPK1a/b with other known RIPK1 proteins in vertebrates. Important sites are circled by red squares. DL/FG can serve as a binding site for Nec-1. I/VQIG plays an important role in mediating protein-protein interactions with RHIM domains. [file Image_2.TIF]

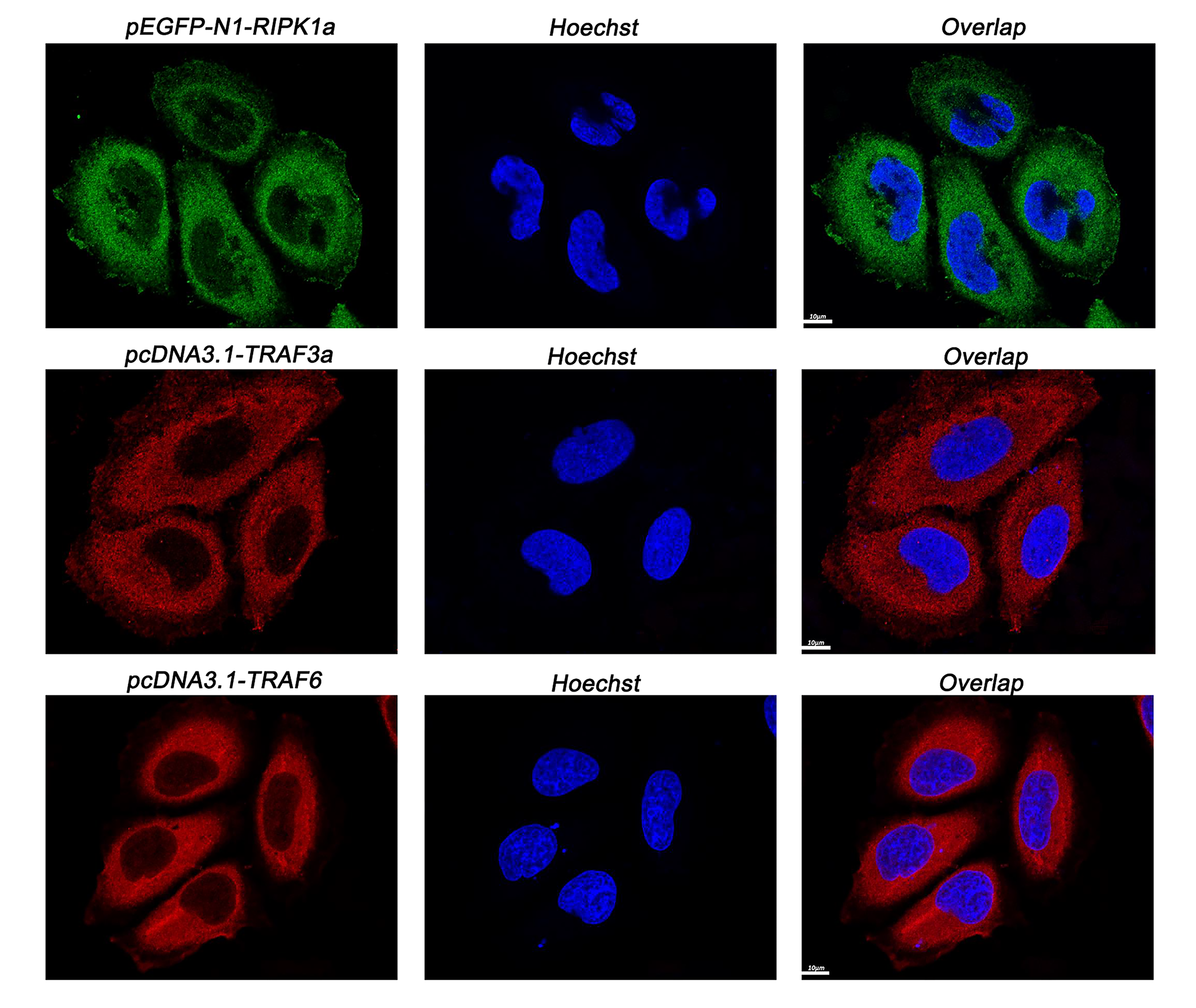

Supplement: Figure S3 — Immunofluorescence microscopy images of HeLa cells transfected with GFP fusion LjRIPK1a, HA-tagged pcDNA3.1-LjTRAF3a or -LjTRAF6 and stained with anti-HA and Annexin-V Alexa Fluor 555 Ab. Data are representative of at least three independent experiments in which >80% of the cells showed similar staining patterns. [file Image_3.TIF]

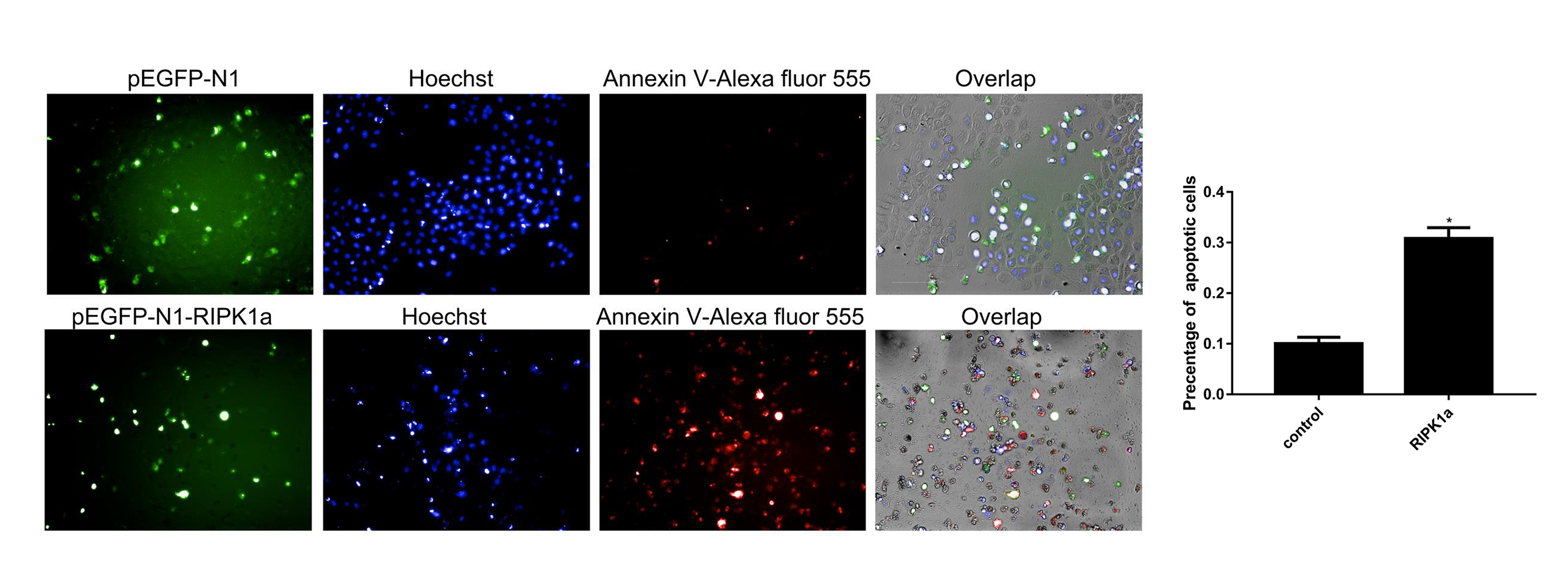

Supplement: Figure S4 — Overexpression of LjRIPK1a leads to apoptosis. All reporter assays were performed in triplicate and repeated with three separate experiments. Values are expressed as the mean fold induction ± SD relative to that of the empty vector control from one representative experiment. [file Image_4.TIF]

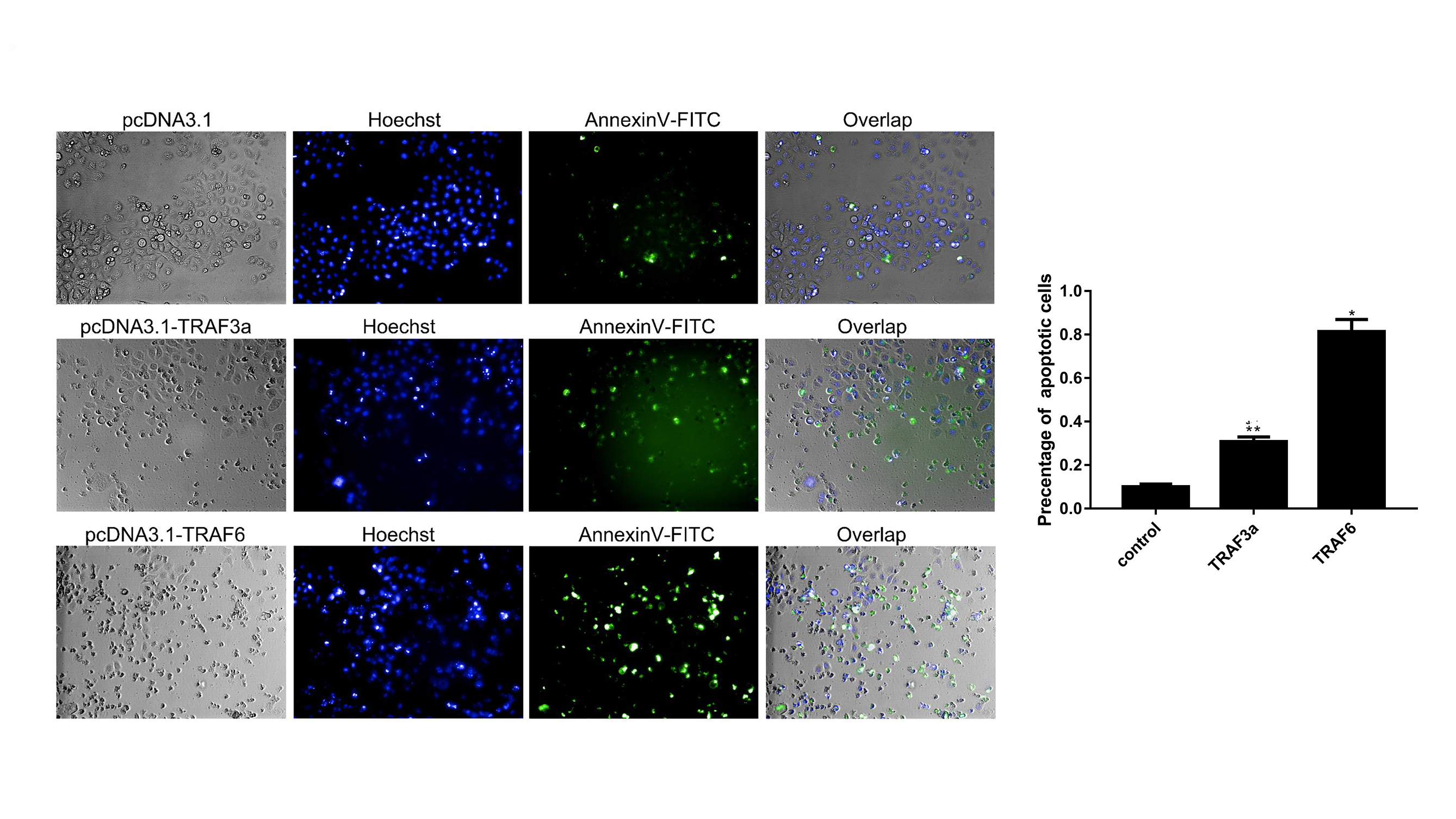

Supplement: Figure S5 — Overexpression of LjTRAF3a/6 leads to apoptosis. All reporter assays were performed in triplicate and repeated with three separate experiments. Values are expressed as the mean fold induction ± SD relative to that of the empty vector control from one representative experiment. [file Image_5.TIF]
