# Supplementary material for: Comprehensive Evolutionary Analysis of Lamprey TNFR-Associated Factors (TRAFs) and Receptor-Interacting Protein Kinase (RIPKs) and Insights Into the Functional Characterization of TRAF3/6 and RIPK1
Source: Front Immunol. 2020 Apr 15;11:663. doi: 10.3389/fimmu.2020.00663 (PMC7179693; doi:10.3389/fimmu.2020.00663)
Supplement: Table S1 — Primers used in this study. [file Data_Sheet_1.docx]

**Table S1: Primers used in this study.**

| Purpose | Accession number | Primer name | Nucleotide sequence (5’-3’ ) | Length(bp) |
| --- | --- | --- | --- | --- |
| RTPCR | MN764208 | *ripk1a*-F | ATGGAGGAGGGTGGCGAGTTT | 2232 |
|  |  | *ripk1a*-R | CCTGGAGACCCCAAGATAAGT |  |
|  | MN764209 | *ripk1b*-F | AGTCACAGAGCAAGCACACACAGC | 1629 |
|  |  | *ripk1b*-R | AGCAGCAGCAGAACGAGGGTCATC |  |
|  | MN764222 | *traf3a*-F | AGCCAGGCAGCAGGCAGCAAG | 1692 |
|  |  | *traf3a*-R | AGGCTTCATCCTCCTTCCACG |  |
|  | MN764223 | *traf6*-F | ATGATCCGCCGCATTCCGACAGC | 1767 |
|  |  | *traf6*-R | AACCACAACCACCACCACCACCA |  |
| qRTPCR | MN764211 | *ripk1a*-F | ATGGAGGAGGGTGGCGAGTTT | 147 |
|  |  | *ripk1a*-R | GCGGTCAAAGTCGTGGGAGAT |  |
|  | MN764212 | *ripk1b*-F | ACGGTAGCCAGCCCCAATCC | 177 |
|  |  | *ripk1b*-R | CGCCTCCTCCTTTGCCTTGTTC |  |
|  | MN764215 | *ripk2*-F | ACGGTAGCCAGCCCCAATCC | 67 |
|  |  | *ripk2*-R | CGCCTCCTCCTTTGCCTTGTTC |  |
|  | MN764213 | *ripk3-F* | AGAGGCACTGGAGAACCGATGG | 81 |
|  |  | *ripk3-R* | GTGCTGCGTGGCTGGACATG |  |
|  | MN764214 | *ripk5*-F | GCCTCGACCTGTTCATCAGC | 80 |
|  |  | *ripk5*-R | TACTGCTCCTCCTCCTTGGC |  |
|  | MN764221 | *ripk7*-F | CGGCATCGCACGGCACTG | 234 |
|  |  | *ripk7-*R | TCAGCAGGTCGTAGAGCAGGAG |  |
|  | MN764224 | *traf3a*-F | GGCACCACAAGGAGGAGTGT | 132 |
|  |  | *traf3a*-R | AGGTGCACCCCAGCATGT |  |
|  | MN764226 | *traf6*-F | CCAACCTCATCTGGCCTTGT | 130 |
|  |  | *traf6-*R | GGATCGGCATCCATCATTTC |  |
|  | MN764227 | *traf7a*-F | CTACAGCGGCTCCTACCAGACC | 191 |
|  |  | *traf7a*-R | AGGTGACGGCGATGGAGTAGAC |  |
|  | MN764228 | *traf7b*-F | TGCCCTCCTGCCGCTTCG | 132 |
|  |  | *traf7b*-R | GCCGTGCGTGTCCGTGTG |  |
|  | KU041137 | *gapdh*-F | AACCAACTGCCTGGCTCCT | 83 |
|  |  | *gapdh*-R | GTCTTCTGCGTTGCCGTGT |  |

**Table S2. GenBank accession number for lamprey sequences submitted in this study.**

| Species | Genes | Accession number |
| --- | --- | --- |
| *Lampetra japonicum* | RIPK1a | MN764208 |
|  | RIPK1b | MN764209 |
|  | RIPK3 | MN764210 |
|  | TRAF3a | MN764222 |
|  | TRAF6 | MN764223 |
| *Lethenteron reissneri* | RIPK1a | MN764211 |
|  | RIPK1b | MN764212 |
|  | RIPK3 | MN764213 |
|  | RIPK5 | MN764214 |
|  | RIPK2 | MN764215 |
|  | TRAF3a | MN764224 |
|  | TRAF3b | MN764225 |
|  | TRAF6 | MN764226 |
|  | TRAF7a | MN764227 |
|  | TRAF7b | MN764228 |
| *Petromyzon marinus* | RIPK1a | MN764216 |
|  | RIPK1b | MN764217 |
|  | RIPK2 | MN764218 |
|  | RIPK3 | MN764219 |
|  | RIPK5 | MN764220 |
|  | RIPK7 | MN764221 |
|  | TRAF3a | MN764229 |
|  | TRAF3b | MN764230 |
|  | TRAF6 | MN764231 |
|  | TRAF7a | MN764232 |
|  | TRAF7b | MN764233 |
|  | TRAF-like | MN764234 |

**Table S3. Conserved motifs discovered among the amino acid sequences of the TRAFs from vertebrates using the MEME system.**

| MOTIF ID | WIDTH |  | BEST POSSIBLE MATCH |
| --- | --- | --- | --- |
| 1 | 41 |  | NGDGMGKGTHLSLFFVVMRGEYDALLPWPFKQKVTFMLLDQ |
| 2 | 29 |  | AVTGRTVSIYSPAFYTGKYGYKMCARAYL |
| 3 | 19 |  | HIIDAFRPDPNWSSFQRPT |
| 4 | 29 |  | PPEDKYKCPICHMVNPVQTPCGHRFCR |
| 5 | 39 |  | DAVPKCPVDNEILDYNKIFPDNFAKREILSLPVYCINEG |
| 6 | 21 |  | EAASYNGIFIWKITDYKRKKQ |
| 7 | 21 |  | IRKRTYVKDDTIFIKCIVDTT |
| 8 | 21 |  | CPFKEYGCTFKGNRNKMARHE |
| 9 | 30 |  | NKCGAMMMRKDLADHLEQECPYRTVTCPYC |
| 10 | 15 |  | GEMNIASGCPLFVPH |
| 11 | 50 |  | DENQSKLSEDLMEFRRDASMLNDELSHINARLNMGILGSYDPQQIFKCKG |
| 12 | 50 |  | FVGHQGPVWCLCVYSMGDLLFSGSSDKTIKVWDTCTTYKCQKTLEGHDGI |
| 13 | 15 |  | HDYDCPYYPVPCPNN |
| 14 | 41 |  | RVWSMDNMICTQTLERHQGSVTALAVSRGHLFDGAVDETVK |
| 15 | 50 |  | YQTIKIWDIRTLECIHVLQTSGGSVYSIAVTNHHIVCGTYENLIHVWDIE |
| 16 | 50 |  | IRAHDNPVCTLVSSHNMLFSGSLKAIKVWDIVGTELKLKKELTGLNHWVR |
| 17 | 29 |  | QNLEERIHELQNRLVQKDQQIQFLHNMMG |
| 18 | 50 |  | FNVIPCPNRCKLSRRDLPAHLQHDCPKRRVKCEFCGCDFTGEAYESHQ |
| 19 | 49 |  | RCALTSEKCPVDNAKLTVVVNNIAVAEQIGELFIHCKYGCRPAASGKPG |
| 20 | 15 |  | KMILMHLQDHLKTCE |

**Table S4. Conserved motifs discovered among the amino acid sequences of the RIPKs from vertebrates using the MEME system.**

| MOTIF ID | WIDTH | BEST POSSIBLE MATCH |
| --- | --- | --- |
| 1 | 29 | PPLLHRDLKPQNILLDKHYHVKIADFGFA |
| 2 | 21 | PWPFRIIHEIAEGMNYLHC |
| 3 | 15 | HDVYSFGIVIWYICT |
| 4 | 29 | WQLMQKCWDQNPDKRPTFQECTTEPFY |
| 5 | 11 | GTPIYMAPEHF |
| 6 | 11 | GGFGTVYKCRH |
| 7 | 28 | RERMELLEEAKIMHMAKFRYIVPVYGIC |
| 8 | 29 | QKEPFENCINIDHIMYCVVQGHRPDRPPI |
| 9 | 21 | EPYGLVMEYMENGSLMKLLHR |
| 10 | 50 | AFACSNRQLEAGHSGRLEMTEDYWVRKDHAPRKARQSLESRDLQDVEF |
| 11 | 50 | RENLGKQWKHCARKLGFTESQIEEIDHDYERDGLKEKVYQMLQKWIMREG |
| 12 | 50 | RTHIACQHGQENIVRILRGVDVNIKGKDDWLPLHYAAWQGHLPIVK |
| 13 | 50 | QAFDMQRDLQITPKRLEYTRKKENELYESLMNIANRKQEEMKDMIVETLN |
| 14 | 41 | PAITLEWKRKVNQKDIDQWSASHFAKQICDQFRTRMWISHN |
| 15 | 50 | QDVSVHITSNYLKQILNAAYHVEVTFHSGSSVTRMLWEQIKQIIQRITWV |
| 16 | 50 | GEPVSSKDIKCCIKQIQELIISRLNQAVANKLISSVDYESFVGTLERC |
| 17 | 33 | WGGHFPCALKSVVPPDEKHWNDLALEFHYMRSL |
| 18 | 50 | NVWDFAGPEEFYSTHPHFMTQRALYLVVYDLSKGQAEVDAMKPWLFNIKA |
| 19 | 50 | PLHLAAQRGHYRVARILIDLCSDVNIPNRLLQTPLHVAAETGHTSTARLL |
| 20 | 50 | MKILQPQDVDLVLDGNSSLLHLAVEAGQEECVKWLLLYNANPNLTNKKGS |
